# High‐throughput assessment of anemophilous pollen size and variability using imaging cytometry

**DOI:** 10.1111/nph.70070

**Published:** 2025-03-28

**Authors:** Thomas Hornick, W. Stanley Harpole, Susanne Dunker

**Affiliations:** ^1^ Department of Physiological Diversity Helmholtz‐Centre for Environmental Research (UFZ) Permoserstraße 15 04318 Leipzig Germany; ^2^ German Centre for Integrative Biodiversity Research (iDiv) Halle‐Jena‐Leipzig Puschstraße 4 04103 Leipzig Germany; ^3^ Martin‐Luther‐Universität Halle‐Wittenberg, Naturwissenschaftliche Fakultät I – Biowissenschaften 06099 Halle (Saale) Germany

**Keywords:** imaging flow cytometry, plant trait, pollen grain size, pollen variability, trait variability

## Abstract

- Pollen grain size relates to plant community structure via pollen dispersal, plant resource allocation into regenerative processes, plant phylogeny and plant genetics (ploidy), or it can be used as a decisive trait for pollen species distinction. However, the availability of pollen size data is limited because of labor‐ and time‐consuming methodological constraints and is classically based on fewer than 50 measured pollen grains per species, thus restricting our knowledge of the temporal and spatial variability of pollen size in response to biotic and abiotic conditions.
- We addressed this data gap by using imaging flow cytometry (IFC), which allows for high‐throughput assessment of pollen size and measured > 500 000 single pollen from 100 anemophilous species that were sampled between 2018 and 2022.
- We present a workflow for high‐throughput data analysis, show the agreement of IFC estimates with literature size estimates and assess pollen size variability in the context of plant phylogeny.
- Our approach allows us to make statistically robust measurements of pollen size that are not limited by sampling effort and sample throughput to answer broad ecological questions at large temporal and spatial scales.

## Introduction

Pollen size data are important for community and functional ecology, evolutionary biology, macroecology or paleobotany for distinguishing species, quantifying species abundance, exploring and predicting spatial and temporal species distributions, as well as gaining a detailed understanding of underlying ecological and evolutionary processes (Mäkelä, 1996; Cruden, 2000; Sork *et al*., 2002; Borrell, 2012; Theuerkauf & Couwenberg, 2022; Wei *et al*., 2023). Also, informing the public about allergenic airborne pollen relies on pollen size data that are one parameter for modeling airborne pollen transport (i.e. pollen forecast) (Dbouk *et al*., 2022). Pollen size information can further be used to explore resource allocation between generative and vegetative strategies of plant reproduction (Cruden & Lyon, 1985) or may be applied in agricultural systems to assess the ploidy level of plants and the evolution of breeding systems (Johansen & von Bothmer, 1994). Pollen size estimates may be applied in plant cultivation as a decisive parameter that restricts the dispersal distance of genetically modified organisms or might be used to distinguish between cultivars and wild‐types (Chaturvedi *et al*., 1998; Joly *et al*., 2007; Williams, 2010; Yang *et al*., 2012; Hofmann *et al*., 2014).

However, pollen size data are limited and often do not provide estimates of variation or only provide categorical pollen size ranges (e.g. TRY (Kattge *et al*., 2020), PalDat (2000 onwards, www.paldat.org) and Pollen‐Wiki (https://pollen.tstebler.ch/MediaWiki/index.php?title=Pollenatlas)). Pollen size is mainly quantified by time‐consuming manual measurements using light or scanning electron microscopy, for which the samples are treated and/or embedded, for example, with alcohol, glycerine jelly, silicon oil or acetolysis, which can impact grain size estimates by either shrinkage or swelling (Mäkelä, 1996; Hayat *et al*., 2009; Beug, 2015; Bolinder *et al*., 2015; Halbritter *et al*., 2018; Lu *et al*., 2018, 2022). Usually, 10–50 randomly selected grains are measured for only a limited number of sites and years (Sótonyi *et al*., 2000; Hayat *et al*., 2009; Hall & Walter, 2011; Beug, 2015; Lu *et al*., 2022), thus restricting our knowledge on inter‐ and intraspecific variability and spatiotemporal variation of pollen size.

To examine the size of a much larger number of pollen grains, promising developments have been made using, for example, the Classifynder automated palynology system (Holt & Bebbington, 2014), laser diffraction granulometry (Bell *et al*., 2018) or network‐based tools for analyzing images of automated microscopic systems (Theuerkauf *et al*., 2024). Also, the use of imaging flow cytometry (IFC), which allows for various applications for environmental monitoring tasks (Hofmann *et al*., 2021; Dunker *et al*., 2022), has been demonstrated for high‐throughput imaging of pollen and the usage of these images and image features for automated species identification using convolutional neural networks for a set of 35 zoophilous (i.e. insect‐dispersed) species (Dunker *et al*., 2021) and 53 pollen standards (Barnes *et al*., 2023), respectively.

In contrast to Dunker *et al*. (2021) and Barnes *et al*. (2023), we here used IFC to explore inter‐ and intraspecific variability of pollen grain size of 100 anemophilous plant species in the context of phylogeny that were sampled across different sites and/or years mainly in urban environments within the city of Leipzig. We present a detailed workflow for pollen sampling, sample preparation, IFC data analysis and recommend for a gating strategy and image mask calculation that was used to estimate size features from pollen images but can also be used for general application in trait research to reveal additional image features. Since pollen size might change with various factors during sample processing and measurement, causing shrinkage or swelling as mentioned earlier, we compared the agreement of IFC data with previously published data from different sources that deviate in pollen sample processing protocols and measurement using traditional light microscopy (Fitter & Peat, 1994; Beug, 2015; Kattge *et al*., 2020; Stebler, 2022). Finally, we explored size variation of *Betula pendula* ROTH pollen across years, exemplarily demonstrating the application of IFC for the assessment of intraspecific and even within‐individual pollen size variation. *Betula pendula* ROTH was sampled with the largest number of individual plants in our dataset and is particularly relevant for various fields of palynology and aerobiology (Cariñanos & Marinangeli, 2021; Dbouk *et al*., 2022; Theuerkauf *et al*., 2024).

## Materials and Methods

### Pollen sampling and sample preparation for IFC

Pollen of Central European species was collected at natural field sites within the city and the surrounding area of Leipzig, and the source plants were identified using Müller *et al*. (2021) and Senghas & Seybold (2003). Additionally, we collected pollen from rare European as well as non‐European species at the Botanical Garden of the University of Leipzig. Pollen was sampled at the peak flowering time of each species from several flowers/inflorescences per individual plant across the Years 2018–2022 (Supporting Information Table S1). We ordered commercially available reference pollen of three species from Allergon AB (Thermo Fisher Scientific, Ängelholm, Sweden) and 16 species from BONAPOL (Bonapol, a.s., České Budějovice, Czechia) (Table S1). Pollen samples were processed and measured with an imaging flow cytometer ImageStream® MK II (Amnis subsidiary of Cytek Biosciences B.V., Amsterdam, the Netherlands) according to the protocol in Box 1. Samples were measured using a ×40 objective or in case of pollen with an expected size larger than *c*. 60 μm using a ×20 objective. The instrument used has a specially designed configuration for the analysis of environmental samples including pollen and algae (US020200278300A1/EP000003692357A) (Dunker, 2020; Dunker *et al*., 2021). For each measurement, images from 5000 particles were recorded, or measurements were stopped after 10 min in case of lower particle concentration.

### Image analysis and feature calculation

**Fig. 1:**
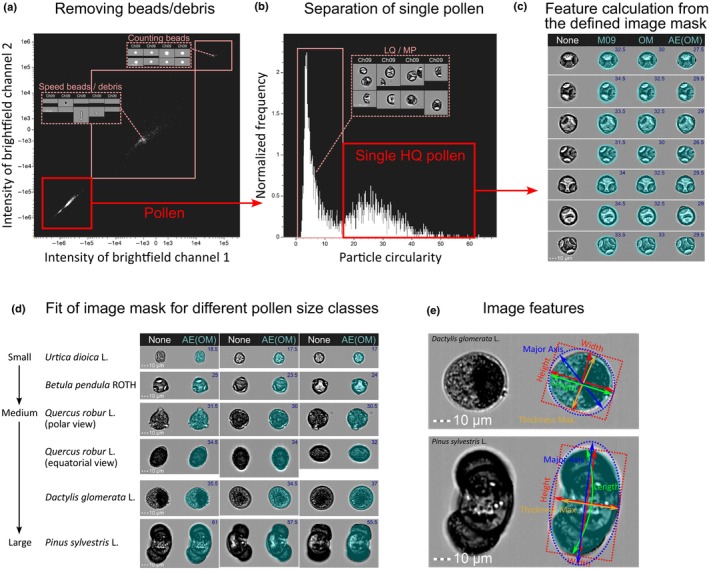
Workflow for image processing using the Ideas cytometry software. (a) First, pollen (*Corylus avellana*) particles were separated from remaining particles including counting beads (optional), speed beads (calibration) and debris using a histogram and/or biplot of brightfield channel intensities from Camera 1 and/or Camera 2. (b) By using a density plot of particle circularity, we presorted single high‐quality (HQ) pollen from single low‐quality (e.g. cropped) and multiple pollen. HQ pollen is defined as an individual pollen grain per image without attachments or remaining debris on the image, which is measurable in all dimensions for proper feature estimation. In this study, HQ pollen was additionally manually inspected to guarantee a high data quality of the analyzed pollen; however, this step might be automatized by standard outlier detection procedures (e.g. to detect pollen with an oversized mask due to additional debris particles on the image). (c) Size features were calculated from HQ pollen based on an ‘AdaptiveErode’‐mask fitted on a brightfield channel (here Ch09) object‐mask (‘AE(OM)’, blue‐shaded area), which best fitted pollen grains (‘M09’, default mask applied on brightfield Ch09; ‘None’, no mask applied to image; ‘OM’, ‘Object’‐mask applied on brightfield Ch09). Numbers in the top right image corner denote the estimated pollen size (‘Length‐feature’) in micrometre for the respective applied image mask, denoting a notable difference between the ‘AE(OM)’‐mask compared with the Default mask ‘M09’. (d) For five species of different pollen size classes, the fit of the ‘AE(OM)’‐mask is indicated, masking the pollen area well including pollen pori, sacci and other pollen sculptures across the whole size range of analyzed anemophilous pollen. (e) Sketch illustrating the difference in size features as implemented in the Ideas software for one rather spherical (*Dactylis glomerata*) and elongated (*Pinus sylvestis* L.) shaped pollen (see text).

Images were collected with the instrument‐specific Inspire software v.200.1.620.1 and processed with the Ideas software v.6.2.187.0. First, debris was removed based on a bivariate plot of brightfield image intensities from the two CCD cameras that are in our instrument settings on Channel 01 and Channel 09 (Ch09) (Fig. 1). In the case of cytometers that are only equipped with one CCD camera, a histogram of one brightfield image intensity can be used for separation instead. Further discrimination of brightfield images of Ch09 with single pollen and multiple pollen was performed by using the circularity feature that measures the degree of particle shape deviation from a circle. Subsequently, manual inspection of all remaining images was performed in the Ideas software in order to estimate high‐quality (HQ) pollen image size features. Manual inspection involved removing other remaining particles, heterospecific pollen, cropped pollen and pollen with debris attachments to yield only single pollen images that allow for unbiased estimation of pollen size features. Six size features were estimated using the Ideas software (‘Length’, ‘Height’ and ‘Width’, ‘Major Axis’, ‘Thickness Max’ and ‘Diameter’), which can be chosen from a set of ‘base features’ that are implemented in the Ideas software and do not require further knowledge on particle feature calculation from images. However, it has to be noted that these features are customized in the Ideas software (Fig. 1e) and might deviate from common practice and/or wording (e.g. see definition of ‘Diameter’). ‘Height’ and ‘Width’ are calculated based on a bounding rectangle and refer to the longer and shorter side, respectively, whereas ‘Major Axis’ defines the longest dimension of an ellipse of best fit. ‘Length’ measures the longest part of an object and can measure an object length even if it is folded. ‘Thickness Max’ measures the largest width of an object. In the Ideas software, ‘Diameter’ defines not a directly measured distance but is calculated as the diameter of a circle that has the same area as the object (therefore not shown in Fig. 1e) (Eqn 1).

(Eqn 1) $$\text{Diameter}=2\times \sqrt{\frac{\text{Area}}{\pi }}$$

The minimum pixel size of the images is 0.5 μm by using a ×40 objective and 1 μm using a ×20 objective, corresponding to the lowest possible resolution of the size feature. Size feature values were exported from the Ideas software as txt‐files and further analyzed using the R‐software.

### Dataset

To eliminate artifacts of sampling on pollen size, we compared the size of our field collected pollen to commercially ordered pollen. The pollen size of field‐collected pollen and commercially ordered pollen was similar ($R_{\mathrm{adj}}^{2}$ = 0.95, df = 14, *P* < 0.0001) (Fig. S1); hence, we included these samples into our dataset. We analyzed 532 736 HQ pollen images derived from pollen of 389 locally sampled individual plants (95 species) and 23 commercially ordered pollen samples (21 species) that include in total 100 species, 46 genera, 23 plant families and 12 plant orders. We calculated the mean and SD of IFC pollen size features at species level (up to 63 681 HQ pollen, mean 5327, median 2247 HQ pollen). Because an uneven sample size of measured pollen of true biological replicates (individual plants) might bias species mean pollen size, we calculated species mean and SD from mean pollen size of individual plants (up to 9433 HQ pollen, mean 1293, median 1006 HQ pollen), which was further used for comparing the agreement of IFC data with literature estimates. In the case of 25 nonspherical species within the genera *Picea* A. DIETR., *Pinus* L., *Carex* L. and *Quercus* L., we compared only size estimates from pollen that was measured in equatorial view (longest dimension) with literature values that also depict this longest dimension. Images of these species that have nonspherical and/or rather oblate pollen were sorted according to polar or equatorial view for feature estimation using a histogram of the ‘elongatedness’ feature and subsequent manual annotation of the remaining images.

For the comparison of IFC size estimates with published data, we used (1) literature size estimations from Beug (2015) including Poaceae data from Rohde (1959), (2) the TRY‐database (Kattge *et al*., 2011, 2020) and (3) the Pollen‐Wiki (http://pollen.tstebler.ch, CC‐BY‐SA 3.0 CH) (Stebler, 2022). However, since the TRY‐database, as one of the largest and most comprehensive plant trait databases, only consists of one pollen size dataset, we refer to the original data from the Ecological Flora Database (Fitter & Peat, 1994) as contributed to the TRY‐database.

### Statistical analysis

We compared IFC size estimates and published microscopic data using a Bland–Altman plot, a parametric approach based on variance and a graphical method for plotting the difference (diff) between the methods against their mean and evaluation of the bias, given as mean difference ($\bar{\text{diff}}$) ± SD of the differences (*s*) (Altman & Bland, 1983; Bland & Altman, 1986). Thus, the Bland–Altman plot prevents the common statistical artifact of related differences by plotting the difference against either value separately (Gill *et al*., 1985). Only after the visual justification of method agreement against the line of identity, correlations (Pearson's *r*, *P* < 0.05) between IFC and published data were used for choosing the best image feature and to rank the agreement of data from different literature sources, due to the known inappropriate use of measuring method agreement using correlation parameters (Bland & Altman, 1986). As such, *r* measures the strength of a relation but not the agreement, since only points lying along the line of equality will have a perfect agreement, but points lying along any straight line will have a perfect correlation. Also, correlation strongly depends on the range of true quantity, and the test of significance is irrelevant to the question of agreement, since it would be surprising if methods designed to measure the same would not be related (Bland & Altman, 1986).

We used R 4.2.0 (https://www.r‐project.org/) and further packages dplyr 1.1.3 (Wickham *et al*., 2022) and ggplot2 3.4.3 (Wickham, 2016) for data analysis and figure preparation. For phylogeny‐based analyses, we used the dated DaPhnE phylogenetic tree with calibrated branch lengths in million years (Durka & Michalski, 2012). We added species that were not present in the tree at the genus level using the ‘phytools::add.species.to.genus’ function, changed tip‐labels of *Elytrigia repens* and *Carex ovalis* to their updated taxonomy *Elymus repens* (L.) GOULD and *Carex leporina* L. and added *Ginkgo biloba* L. and *Parrotia persica* C.A. MEYER to Ginkgoaceae and Hamamelidaceae, respectively. We tested for a phylogenetic signal of pollen size using Blomberg's *K* (Münkemüller *et al*., 2012) using R‐packages ape 5.8 and phytools 2.3‐0. The significance of the observed phylogenetic signal was determined using randomization (*n* = 1000).

## Results

### Choice of image mask and IFC size feature

All features are based on an input mask, which is the set of pixels that contain the region of interest. An ‘Adaptive Erode‐mask’ with a threshold value of 95 on the ‘Object‐mask’ of Ch09 best fitted the shape of pollen grains on the generated brightfield images across pollen of different size and orientation and was specified using the available set of masks as implemented in the Ideas software by visual inspection (Fig. 1c,d). Nonetheless, visual inspection alone might not be suited to proof how accurate size measurements with the IFC approach are. IFC does not allow to sort or trace individual pollen for comparing their size with a different method (e.g. microscopy). Even if this would be possible, pollen size might be variable due to the orientation of pollen, sample preparation or mounting. Therefore, we determined the accuracy of IFC size estimates and explored the magnitude of the expected effect on size estimations by different mask settings using certified Latex beads traceable to NIST Standard with a modal diameter of 19.98 μm (Coulter® CC Size Standard L20; Coulter Corp., Miami, FL, USA). Because the calculation of the six size features might be sensitive to the input mask shape that fits the shape of pollen and thus deviates from a perfectly round mask fitting beads, we evaluated additionally the most appropriate size feature for pollen grains by evaluating their agreement with published size data (Table S2). Generally, all six IFC size features agree with literature values against the line of identity. ‘Diameter’, ‘Height’, ‘Length’ and ‘Major Axis’ might be considered equivalent as a measure of pollen size for the anemophilous species in our dataset (Pearson's *r* = 0.91–0.97). Although for round‐shaped pollen, the six size features might result in similar estimates, features will deviate for oblate or elongated pollen; for example, the ‘Diameter’ feature is highly dependent on a well‐fitting mask and only a proper choice for round particles (Fig. 1e). From all six size features, we selected the ‘Length’ feature as most robust feature that measures the longest part of an object in micrometre (Fig. 1e). The ‘Length’ feature of the pollen adapted ‘Adaptive Erode‐mask’ with a threshold value of 95 on the ‘Object‐mask’ of Ch09 accurately resembled modal size of the L20 latex beads (Fig. S2). By contrast, the ‘Default masks’ of brightfield images overestimate modal size of the L20 beads by 6 μm using a ×20 objective (*n* = 5565) and 4.5 μm using a ×40 objective (*n* = 6522).

### Agreement of IFC pollen size data with microscopy‐based literature estimates

**Fig. 2:**
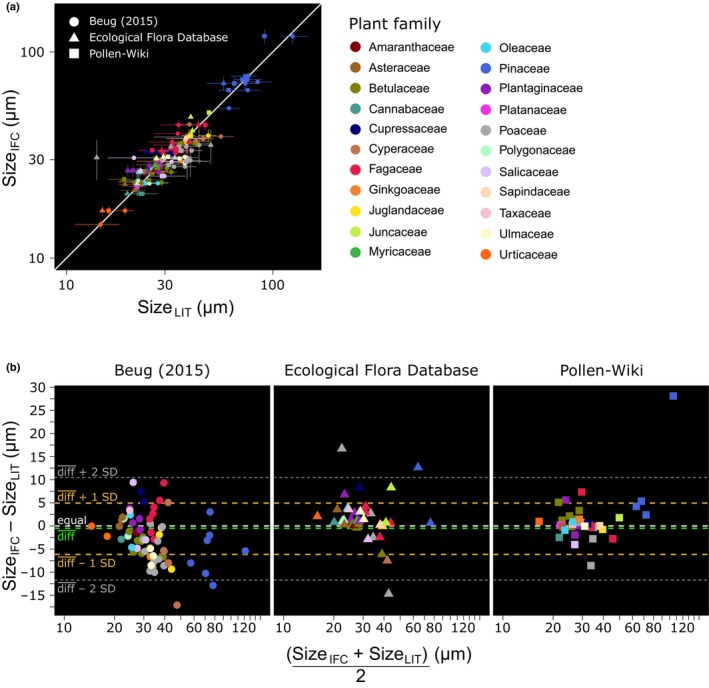
Agreement between pollen size data measured with imaging flow cytometry and literature values. (a) Comparison per species of mean pollen size (1 SD) measured with imaging flow cytometry (IFC) (sizeIFC) and literature values (sizeLIT) provided as minimum, mean and maximum values from (Beug, 2015) (dots), the Pollen‐Wiki (Stebler, 2022) (squares) and the Ecological Flora Database (Fitter & Peat, 1994) as contributed to TRY (Kattge *et al*., 2011, 2020) (triangles). Line of equality: white solid line. Since most pollen grain size ranges from 20 to 50 μm, axes were log_10_‐transformed for better visualization of the highly clustered point space. (b) A Bland–Altman plot that visualizes the difference (diff) between IFC and literature size estimates from three literature sources against the mean of both estimates per species. Lines denote equality (overall zero diff (white), mean‐diff (diff, green), diff 1 SD (yellow) and diff 2 SD (gray)). Point colors denote the phylogenetic association of a species to a plant family.

When comparing the agreement of IFC size data with microscopy‐based literature estimates from different sources, we have to consider that no method may provide an unequivocally correct measurement due to methodological artifacts each method has; thus, the true value to compare with is basically unknown. We assessed the degree of agreement by comparing against the line of identity (Fig. 2a) and used a Bland–Altman plot to visualize the diff between IFC and literature size estimates against the mean of both estimates (Fig. 2b). The smallest $\bar{\text{diff}}$ across species and best agreement with literature estimates suggested by the goodness of fit of a linear model was obtained between IFC data and data from the Pollen‐Wiki (size_IFC_ = 1.0 × log_10_(size_LIT_) + 0.006; $R_{\mathrm{adj}}^{2}$ = 0.93; df = 36; *P* < 0.0001). The lowest goodness of fit between IFC and literature data was observed with data from the Ecological Flora Database ($R_{\mathrm{adj}}^{2}$ = 0.76) and is driven by larger diff of wild grass pollen (Poaceae) size.

We further used the Bland–Altman plot to explore systematic patterns in diff across the size gradient of pollen (e.g. between sets of small and large pollen) or patterns that relate to the phylogenetic association of pollen species. The Bland–Altman plot suggests a homogeneous distribution of diff across the size range of measured pollen, in which most species range within $\bar{\text{diff}}$ ± *s* = −0.5 ± 5.5 μm. Patterns in diff that correspond to the phylogenetic association of pollen are not consistent across published data and specific to the distinct data sources, suggesting consistent differences in pollen size due to sample preparation (embedding medium), measurement and/or sample origin (size variability in response to environment) and/or might reflect unexplained variability. Beug (2015) arrived at larger sizes for Poaceae (−5.7 ± 3.6 μm, *n* = 12), Cyperaceae (−6.7 ± 7.9 μm, *n* = 5), Betulaceae (−4.1 ± 1.9 μm, *n* = 8) and Ulmaceae (−6.6 ± 1.8 μm, *n* = 3) and smaller sizes for Juncaceae (4.5 ± 5.4, *n* = 2) and Cupressaceae (6.4 ± 1.6, *n* = 2) compared with IFC estimates. Betulaceae pollen measured with IFC is similar or slightly smaller in size than data from the Ecological Flora Database (−1.0 ± 2.8 μm, *n* = 5) and data from the Pollen‐Wiki (1.8 ± 1.8 μm, *n* = 8). The size of Poaceae pollen (wild grasses) is generally highly variable between datasets. In the case of Pinaceae, Beug (2015) reports larger pollen sizes compared with IFC measurements (−5.5 ± 5.4 μm, *n* = 7), whereas other literature sources report considerably smaller sizes (Ecological Flora Database: 6.6 ± 8.5 μm, *n* = 2; Pollen‐Wiki: 10.0 ± 12.1 μm, *n* = 4).

### IFC as a tool to assess inter‐ and intraspecific pollen size variability

**Fig. 3:**
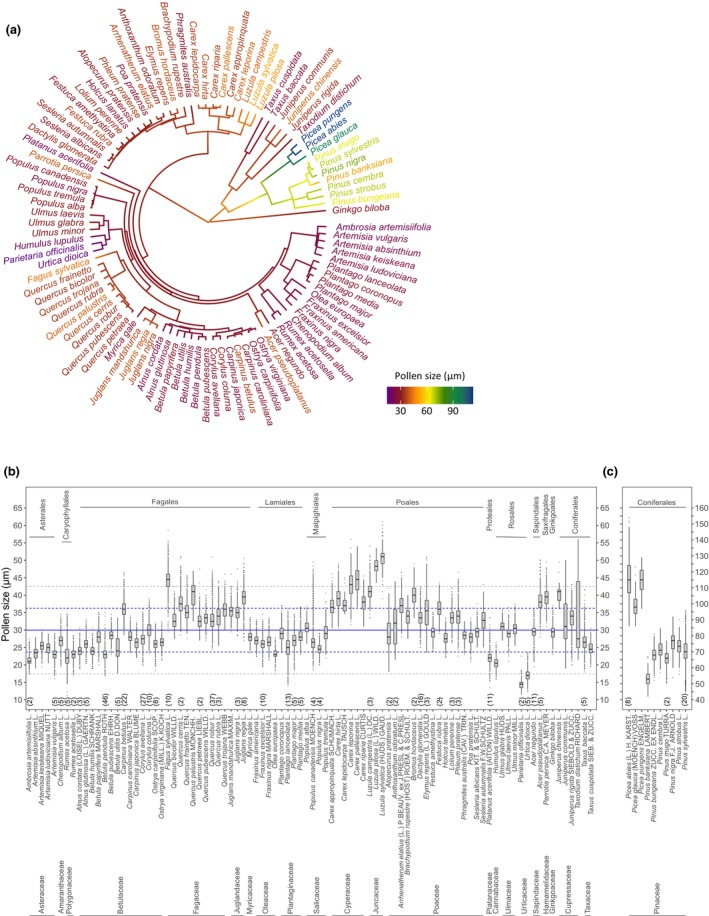
Anemophilous pollen size in context of plant phylogeny and taxonomy. (a) Mapping of pollen size (species mean of ‘Length’ feature) to the phylogeny of the 100 investigated anemophilous plant species. (b, c) Boxplots visualizing the whole observed variability of pollen size (‘Length’ feature) of all measured pollen. Species are sorted according to plant taxonomy (order and family level). Note different axis for (b) nonsaccate pollen and (c) saccate pollen. The solid blue, dashed blue and dashed gray lines denote species mean, mean 1 SD and mean 2 SD pollen size for nonsaccate pollen, respectively. Numbers in brackets denote the number of individual plants from which pollen was sampled (otherwise only one individual plant was sampled). Further information (e.g. the number of pollen and years) can be obtained from Supporting Information Table S1. In boxplots, the thick horizontal line represents the median, hinges the first and third quartiles (interquartile range (IQR)) and whiskers extend to the 1.5 IQR.

The size of pollen estimated by IFC in our study ranged from 14.5 ± 0.8 μm (*Parietaria officinalis* L.) to 119.1 ± 9.2 μm (*Picea abies* (L.) H. KARST) (Fig. 3). Saccate (i.e. vesiculate) Pinaceae pollen is substantially larger in size than pollen from other plant families and has the largest size variability of the studied plant families (genus *Pinus*: 68.2 ± 7.5 μm; genus *Picea*: 111.1 ± 10.5 μm). Pinaceae pollen form two air sacs as an exinous expansion to the monad pollen corpus and are therefore morphologically distinct from all other nonsaccate pollen (Fig. 1). By mapping pollen size to phylogeny to test for a phylogenetic signal across the set of the 100 anemophilous species, Blomberg's *K* showed a strong significant phylogenetic signal (*K* = 1.30, *P* = 0.001), indicating that closely related species are more similar in pollen size than would be expected by random evolution. As indicated by comparing Blomberg's *K* including (*K* = 1.30, *P* = 0.001) and excluding (*K* = 0.25, *P* = 0.001) Pinaceae from phylogeny, this phylogenetic signal is mainly driven by Pinaceae. Although the signal remains significantly different from what could be expected under a model with no phylogenetic signal, the pollen size of nonsaccate species might be more influenced by ecological factors or convergent evolution and not be strongly conserved across related species, as indicated by a low *K* value. As such, substantial size differences can be observed for nonsaccate pollen between *Carpinus betulus* L. compared with *Carpinus caroliniana* WALTER and *Carpinus japonica* BLUME, between *Fagus sylvatica* L. and *Quercus* species, and between *Acer negundo* L. and *Acer pseudoplatanus* L., but also for saccate pollen between *Pinus bungeana* ZUCC. EX ENDL. and other *Pinus* species in which pollen size differentiates substantially between species at the genus and/or family level (Fig. 3). These differences might represent forms of environmental adaptation, for example climatic and geographical adaptation of species that naturally occur on different continents; however, further statistically supported exploration needs measurements from multiple individual plants and populations, which are not yet covered by our dataset. Sizes of the 90 anemophilous nonsaccate pollen species range from 14.5 μm (*P. officinalis* L.) to 50.8 (*Luzula sylvestris* (HUDS.) GAUD.) with a mean (±1 SD) species average pollen size of 30.9 ± 6.7 μm. Nonsaccate pollen size varies between plant families up to 2.9‐fold (15.8 ± 1.7 μm (Urticaceae)–46.8 ± 4.9 μm (Juncaceae)) with an interspecific size variability of up to 12.6 μm within a plant family (Betulaceae).

**Fig. 4:**
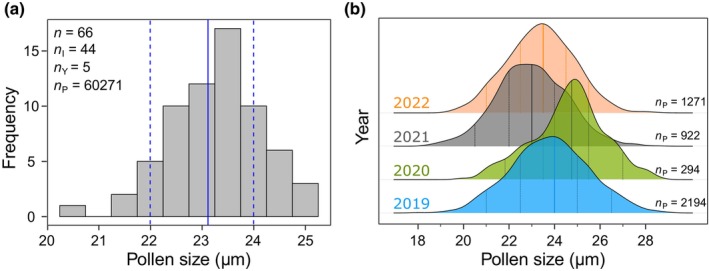
Intraspecific and within‐individual pollen size variation of *Betula pendula* ROTH. (a) Frequency of observed mean pollen size (‘Length’ feature) of *Betula pendula* ROTH that was calculated for each individual tree per year (*n*). The blue lines denote the mean size (solid) and the interquartile range (25^th^ and 75^th^ percentile, dashed) of all measured pollen. *n*_I_, number of individual plants; *n*_P_, number of pollen; *n*_Y_, number of years. (b) Shown exemplarily for one *B. pendula* ROTH tree, imaging flow cytometry can be used as a tool for analyzing within‐individual plant pollen size variation across years, for example to explore variation by climate and habitat. Lines denote 5^th^, 25^th^, 50^th^ (median), 75^th^ and 95^th^ percentiles.

We estimated how much intraspecific and within‐individual variability in pollen size can be expected in the case of *Betula pendula* L. pollen, the species that was sampled with the largest number of individual trees in our dataset. From the 46 individual trees that were sampled overall, 18 trees were sampled across several years. The mean pollen size, calculated for individual trees per year (*n* = 66), ranged from 20.5 to 25.0 μm (mean = 23.29 ± 0.89 μm) (Fig. 4a). Within‐individual variability of mean pollen size was up to 2.65 μm (mean = 0.99 ± 0.72 μm) between years (Fig. 4b).

## Discussion

Imaging flow cytometry measurements could be demonstrated to be in the same range as previously derived microscopic size measurements from different sources. This is especially interesting because our protocol deviates from traditionally used protocols in sample storage, sample preparation (e.g. embedding medium), time of measurement after sampling and number of measured pollen (e.g. Beug, 2015), and we consider samples with different sample sizes, of different origins and from multiple years. Sample storage and the medium in which the pollen is situated at the moment of measurement is an important factor, affecting pollen size estimation, for example by dehydration or swelling (Mäkelä, 1996). Therefore, we took care of applying the same optimized sampling and storage protocols for all samples to ensure measuring matured pollen (Box 1).

Also, laser diffraction granulometry can achieve a higher throughput of millions of measured pollen, but in contrast to IFC, it does not allow to quality‐control single pollen grains by using images, for example for separating heterospecific from conspecific pollen or dirt and other particles of the same size within a sample (Bell *et al*., 2018). Other automated and/or high‐throughput imaging methods use acetolyzed samples, need to include additional segmentation steps and deal with overlapping pollen or do not report on the fit of the mask that was used during image analysis that might largely impact size estimation, as shown in this study (Allen *et al*., 2008; Holt & Bebbington, 2014; Theuerkauf *et al*., 2024). Barnes *et al*. (2023) heated pollen to 90°C before IFC measurement to increase the autofluorescence of pollen; however, this strong heat treatment can alter morphological features. It might be noted that the size of pollen measured in air might also differ from pollen embedded in a liquid for measurement, which can be relevant for pollen dispersal models.

Some small and consistent discrepancies in pollen size for specific plant families between IFC measurements and literature estimates might relate to differences in aging, sample preparation and dehydration. Pollen reported by Beug (2015) was treated by acetolysis (acetic anhydride and sulfuric acid; 10 : 1) and embedded in glycerine–gelatine according to Kisser, which both can cause swelling of pollen (Mäkelä, 1996) and might correspond to the slightly larger pollen sizes observed for most plant families compared with IFC data for this dataset. Interestingly, pollen sizes reported by Stebler (2022) show the opposite trend (e.g. slightly smaller pollen of Betulaceae), although the pollen was treated in a similar way with acetolysis and embedding pollen in glycerine–gelatine according to Kaiser, suggesting other factors such as the time of sampling during the flowering period, genetic variation, environmental conditions at a plants’ location or unexplained variation causing the observed differences in pollen size. As such, ploidy and nutrients have been reported to contribute to differences in pollen size (Johansen & von Bothmer, 1994; Lau & Stephenson, 1994).

We observed large intraspecific and within‐individual variation, although our data only capture a small spatial gradient around the city of Leipzig. Albeit only exemplarily shown for *Betula pendula* ROTH, for which we sampled most true biological replicates (individual trees), intraspecific pollen size varied largely by up to 4.5 μm (mean value) between individuals and years. Even within‐individual variability between years was up to 2.6 μm (mean value), which is in the order of size difference that was used to discriminate between *Betula* L. species in former studies (Mäkelä, 1996; Theuerkauf *et al*., 2024).

Since the method is now at hand, it would be interesting to explore inter‐ and intraspecific variation in pollen size further across larger spatial and temporal gradients and climatic zones to study how explanatory biotic and abiotic variables relate to pollen size (Wei *et al*., 2024; Walther *et al*., 2025). In that context, it is particularly interesting that the lowest goodness of fit was observed between IFC estimates and data from the Ecological Flora Database representing data from Great Britain that might also represent the largest difference in environmental conditions between sampling sites of the analyzed datasets.

In addition to the environment, a strong evolutionary constraint on anemophilous pollen size could be not only adaptation to wind‐dispersal (Ackerman, 2000; Culley *et al*., 2002; Friedman & Barrett, 2009) but also the matching female flower traits that might relate to the magnitude of observed interspecific variation across phylogenetic groups. Although we did not analyze pollen size in the context of other plant traits in this study, we tested for a phylogenetic signal in which a strong signal might suggest coevolution of pollen size and floral or vegetative plant traits that are distinct for certain phylogenetic groups. We could only detect a strong phylogenetic signal for Pinaceae across the set of 100 anemophilous species. The sacci of Pinaceae have been reported to play an adaptive aerodynamic role (Schwendemann *et al*., 2007; Grega *et al*., 2013); however, the ‘floating hypothesis’ by Leslie (2010) in fact reveals their functional role to float upwards in a liquid pollination droplet towards the downward‐facing ovule that is a strong evolutionary constraint in Pinaceae and different from the other anemophilous species (Halbritter *et al*., 2018). By contrast, only a weak phylogenetic signal was present for nonsaccate species, confirming the results of Wei *et al*. (2023) who also detected only a low phylogenetic signal for Poaceae. The observed low variance in nonsaccate pollen size across species compared with the variance expected by a Brownian motion model suggests a larger importance of adaptive evolutionary processes that might be adaptation to a certain size range that is optimized for wind‐dispersal (Ackerman, 2000; Culley *et al*., 2002; Friedman & Barrett, 2009) or environmental conditions that should not vary much within the here‐sampled spatial scales.

In this study, we focused on anemophilous (i.e. wind pollinated) species and adapted our workflow to separate HQ pollen images from remaining images based on bivariate plots of selected image features, using ‘circularity’ as a discriminating image feature. Although, to our knowledge, this workflow fits most pollen, it might be adapted to specific pollen types that largely deviate from the here‐analyzed mostly spheroid pollen types in their shape (e.g. for adapting a gating strategy fitting zoophilous pollen species). In addition, flawed or inaccurate masks can have a significant impact on the estimation of overall image features (Dominical *et al*., 2017); thus, great care was taken to ensure the accuracy of the mask by using standardized beads to best fit the pollen area on an image across the size range of analyzed anemophilous pollen ranging from Urticaceae to Pinaceae pollen (Fig. 1).

Entomophilous pollen was not specifically part of this work. However, these pollen grains encounter an even greater variability in pollen sizes and shapes. Therefore, when developing suitable gating strategies as well as for identifying appropriate masks, great care has to be taken to apply the most suitable mask and derive microscopy‐related size traits, especially for noncircular pollen. In that case, polar and equatorial views also need to be considered when deriving size traits. Size properties of fossil pollen can principally be derived in the same way as described here, but species size properties differ in contrast to recent pollen (Barnes *et al*., 2023). However, the described method also allows high‐throughput analysis of fossil pollen to increase spatial data coverage (Dunker *et al*., 2022).

Compared with the usually not > 50 microscopically measured pollen grains, based on some hundreds or thousands of individually measured pollen grains per sample, we are likely to decrease SE and confidence interval widths, increasing the chance of finding statistically significant differences even with a tiny effect size, for example for exploring within‐individual pollen size variation across years (Fig. 4). Thus, with the large sample size, inter‐ and intraspecific variation and biological significance of group differences can be assessed with a yet‐unprecedented accuracy and statistical power. Nonetheless, we want to encourage that such high‐throughput methodologies need to fulfill certain data standards regarding data quality and data management in order to be used in routine monitoring (Hornick *et al*., 2022; Moore *et al*., 2024). For that reason, our workflow includes a manual inspection of pollen grains in order to guarantee a high data quality of our dataset. However, this manual step for data quality control might be replaced by automated protocols for outlier detection or machine learning/deep learning models (Dunker *et al*., 2021) that can adequately replace this task in the future.

### Conclusion

We show that IFC can be used as an accurate and good alternative approach to microscopy for measuring pollen size, while providing the advantage of a high‐throughput method. Thus, our approach can strongly improve spatial and temporal pollen size assessment relevant for community and functional ecology, aerobiology, evolutionary biology, macroecology or paleobotany. The high‐throughput method allows for a detailed analysis of pollen size and its variability not possible with standard methods, thus improving pollen forecast models and biodiversity monitoring by filling a data gap in biodiversity research and allowing for the exploration of large‐scale patterns in species distribution and trait evolution in relation to environmental variables. Albeit we particularly focused on the measurement of pollen size, additional features can also be extracted from pollen images (e.g. shape, texture and multispectral fluorescence intensities), allowing for easy adaptation of the here presented workflow for general trait research.

## Competing interests

The patent submission EP000003692357A1/US020200278300A1 by SD could create a potential conflict of interest, but to date, no financial benefit has been accrued from the patent submission. A cooperation contract between UFZ and Cytek (Amsterdam, the Netherlands) exists, regulating beta‐testing of products.

## Author contributions

TH identified plants and sampled pollen material, prepared them for analysis and performed the imaging flow cytometry analysis. TH and SD did quality‐control of the flow cytometry data and annotated the images. TH analyzed the data, prepared the figures and wrote the first version of the manuscript with support from WSH and SD. All authors helped to shape the research and contributed to the final version of the manuscript. SD supervised the project.

## Disclaimer

The New Phytologist Foundation remains neutral with regard to jurisdictional claims in maps and in any institutional affiliations.

Box 1 **Protocol for anemophilous pollen sampling, sample preparation and measurement using imaging flow cytometry (IFC)**

**Materials**

1. **Reagents**
  - **Pollen sample** (freshly collected, frozen or purchased material)
  - **Reagents for pollen sample processing for cytometric measurement**
    ○ Pollen isolation buffer (PIB) (see the Reagent set‐up section)
      ▪ KH_2_PO_4_ (e.g. cat. no. 1648; Th. Geyer GmbH & Co. KG, Renningen, Germany) [Correction added on 24 April 2025, after first online publication: the details listed in this bullet point have been amended.]
      ▪ EDTA (e.g. cat. no. 131669; AppliChem GmbH, Darmstadt, Germany)
      ▪ Triton X® 100 (e.g. cat. no. 3051.3; Carl Roth GmbH & Co. KG, Karlsruhe, Germany)
    ○ Dulbecco's Phosphate Buffered Saline (DPBS) 1× w/o calcium w/o magnesium (e.g. prepare dilution from 10x solution cat. no. X0515; Biowest, Nuaillé, France)
  - **Flow cytometry reagents**
    ○ Sheath fluid
      ▪ DPBS 1× w/o calcium w/o magnesium (e.g. prepare dilution from 10x solution cat. no. X0515; Biowest, Nuaillé, France)
    ○ Sterilizer
      ▪ Sodium hypochloride 0.6% (e.g. prepare dilution from cat. no. 1305.1000; Th. Geyer GmbH & Co. KG, Renningen, Germany)
    ○ Debubbler
      ▪ 70% 2‐Propanol (HPLC grade)
    ○ Cleanser
      ▪ For example, FlowClean Cleaning Agent (cat. no. C48093; Beckman Coulter Ireland Inc., Lismeehan, Ireland)
    ○ Speed Bead® Image Stream®X System Calibration Reagent (cat. no. 400041; Cytek Biosciences B.V., Amsterdam, the Netherlands )
2. **Equipment**
  - **Materials for pollen sampling and sample processing for cytometry**
    ○ Paper bags (e.g. flat paper bag 130 × 180 + 20 mm, cat. no. 3.130.003; Baumann Saatzuchtbedarf, Waldenburg, Germany) may be used
    ○ Forceps, Petri dishes (10 cm diameter) (for the transfer of plant material, inflorescences, or flowers and the removal of larger plant residuals from pollen material before sieving)
    ○ Drying cabinet (for gently drying of plant materials)
**# RECOMMENDATION** – A drying cabinet should be used with a ventilation system that not only heats but also actively reduces the humidity by ventilation in the chamber; otherwise, the plant material may become moldy. Paper bags are preferred to plastic bags in order to achieve good drying results and avoid electrostatic charging.
**! CAUTION** – Close paper bags carefully to prevent the spread of pollen through ventilation.
    ○ Filter for sieving pollen materia l
**# RECOMMENDATION** – Use filters that fit sample tubes for fast and complete sieving of pollen material directly into sample tubes (e.g. CellTrics® Filter – 30, 50, 100 μm (cat. no. 04‐0042‐2316, 04‐0042‐2313, 04‐0042‐2313), Sysmex Deutschland GmbH, Norderstedt, Germany; Filcon® Filter – 70 μm (cat. no. 12170‐67), Süd‐Laborbedarf GmbH, Gauting, Germany).
**# RECOMMENDATION** – The mesh size should be selected based on the expected pollen size (if known) in order to avoid systematic bias.
    ○ Sample tubes suitable to fit CellTrics® or Filcon® – Filter (e.g. 2.0 ml Eppendorf Safe‐Lock Tubes (cat. no. 0030120094); Eppendorf SE, Hamburg, Germany)
    ○ Sample tubes suitable for flow cytometry (e.g. 1.5‐ml Eppendorf Safe‐Lock Tubes (cat. no. 0030120086); Eppendorf SE)
**! CAUTION** – Only 1.5‐ml sample tubes fit the ImageStream®X MK II flow cytometer (Amnis, subsidiary of Cytek Biosciences B.V., Amsterdam, the Netherlands) in the case of manual mode of measurement. In case of using an auto sampler attached to the instrument, other sample tube sizes may be used.
    ○ Sample tube holder
    ○ Pipette
    ○ Ultrasonic bath
**! CAUTION** – For this protocol, we used an ordinary ultrasonic bath, for example for cleaning glasses (Bandelin Sonorex Digitec DT 31 H, Bandelin electronic GmbH & Co. KG, Berlin, Germany; 35 kHz, 21°C). However, when using an ultrasonic bath, check empirically if pollen might be destroyed by too high acoustic intensities and reduce acoustic intensity accordingly.
    ○ Vortexer
    ○ Centrifuge (e.g. Eppendorf 5425R; Eppendorf SE)
    ○ −20°C freezer for sample storage
  - **Materials for cytometry**
    ○ Imaging cytometer, for example ImageStream®X MK II flow cytometer (Amnis subsidiary of Cytek Biosciences B.V., Amsterdam, the Netherlands)
**# RECOMMENDATION** – When working with a two‐camera ImageStream®X MK II system, a non‐colinear laser alignment is recommended (see also patent publication US020200278300A1/EP000003692357A) (Dunker, 2020).
    ○ Appropriate software for the measurement (instrument‐specific Inspire software (here we used v.200.1.620.1)) as well as processing raw image files (.rif) (Ideas software (here we used v.6.2.187.0)).
3. **Reagent set‐up**
  - **Pollen material**
    ○ Sampling
**# RECOMMENDATION** – Samples should be fresh, ideally sampled from intact flowers or inflorescences. Partly opened or open anthers (or complete parts of inflorescences in the case of tiny anthers) can be transferred with forceps directly into 2‐ml sample tubes to reduce the loss of pollen. We recommend the extraction of pollen immediately after sampling to prevent effects of aging or molding of pollen in the tubes (see the Procedure section).
**Δ CRITICAL** – It is important to choose an appropriate developmental stage of pollen. Note that not fully developed (i.e. unripe), senescent, degraded or otherwise differentiated pollen may deviate in pollen traits from fresh, matured pollen.
**! CAUTION** – In particular, most anemophilous pollen have an allergenic potential and may cause sensitization. Therefore, use personal protective measures: goggles – prevent eye irritation; FFP2 mask – avoid inhalation; disposable gloves – avoid skin contact; use of a fume hood – avoid spread of pollen in the laboratory; strict separation of laboratory clothing from daily clothing – avoid spread of pollen outside laboratory; and wearing of oversleeves – reduce the amount of pollen on clothing. In case of highly allergenic pollen material such as of *Ambrosia artemisiifolia* L., a fullbody suit should be used and be disposed.
    ○ Gently drying of inflorescences and pollen material
**# RECOMMENDATION** – Usage of fresh pollen from ripe and partly opened anthers should be preferred (as described earlier). When a larger amount of pollen material is needed (i.e. to perform additional analysis) or pollen should be stored at room temperature (i.e. prevention of molding), intact inflorescences/flowers with ripe pollen but not yet opened anthers can be sampled. The time of sampling should be as close as possible before natural opening of anthers (*c*. 1 d), which requires extensive (i.e. daily) monitoring of phenology to guarantee collection of fully developed/matured pollen based on observations when first opening of anthers occurs within a local plant population. Flowers/inflorescences are collected into paper bags and dried gently at 30°C in a drying chamber in order to prevent changes on pollen chemistry, physiology and/or pollen morphology due to heat treatment (i.e. degradation of enzyme activity or pigments). The gently drying causes opening of anthers, allowing for complete sampling of released pollen into the paper bags.
**! CAUTION** – Close paper bags carefully to prevent the spread of pollen through ventilation.
    ○ Pollen sieving to remove artefacts (plant debris, insects, etc.).
**# RECOMMENDATION** – Use of filters that fit sample tubes to reduce loss of pollen material. The filters are placed on the sampling tube and pollen is sieved using a gentle tapping motion.
    ○ Storage of pollen samples:
      ▪ Storage at room temperature
**! CAUTION** – Molding must be avoided when storing pollen samples at room temperature, thus storing only dried material (as described earlier). The period for which pollen can be stored might differ between species and should be determined empirically. In any case, materials potentially colonized by pests (fungi, insects and tissues with insect eggs laid on) harbor risk of biological contamination or loss of pollen due to feeding insects, and should therefore be avoided.
      ▪ Storage at −20°C in a freezer
  - **PIB** – 100 mM KH_2_PO_4_, pH 7.5; 1 mM EDTA; 0.1% (v/v) Triton X‐100 (according to Aloisi *et al*., 2015) with the modification of using KH_2_PO_4_ instead of Na_2_HPO_4_.
4. **Equipment**
  - **Flow cytometer:** Check that reagent containers (Sheath, Sterilizer, Debubbler, Cleanser and Rinse Reagent (0.2 μm filtered deionized water)) are filled and waste container is emptied. Run the ASSIST test for calibration of the system (recommended on a daily basis before the start of the measurements). In case of not proper functioning of fluidics or air bubbles and/or blocking particles in the tubing system, the ASSIST test will result in an error.
5. **Procedure**
  - Pollen extraction protocol
    ○ Transfer sieved pollen material (we recommend not to use > 1 mg), a part of an inflorescence or partly open/open anthers into 2.0‐ml sample tubes.
    ○ Add Pollen Isolation Buffer to samples (usually 200–700 μl to cover the sample).
    ○ Place samples for 5 min in an ultrasonic bath to separate pollen and destroy aggregates.
    ○ Vortex samples
    ○ Filter samples (see the Equipment section) into 1.5‐ml sample tubes.
**# RECOMMENDATION** – By pouring the samples directly from the sampling tubes into the filters, a fraction of pollen might still stick to walls of the sampling tubes and the filter. Rinsing sampling tubes a second time with PIB (minimum 200 μl) reduces pollen loss during sample preparation.
    ○ Centrifuge samples (4000 ***g***, 2 min) and remove supernatant.
    ○ Add DPBS to the pollen pellet and vortex (usually 50–100 μl DPBS is added).
  - We recommend immediate measurement of prepared pollen samples. Otherwise, store prepared pollen samples at −20°C until measurement.
  - Measurement
    ○ Settings used for this protocol
      ▪ 488 nm laser intensity: 5 mW
      ▪ 561 nm laser intensity: 20 mW + neutral density filter (ND 1.0)
      ▪ 785 nm laser intensity: 0.1 mW
      ▪ Magnification: ×40 objective recommended for pollen < 60 μm, ×20 objective recommended for pollen > 60 μm.
**! CAUTION** – The upper size limit depends on the used filter size during sample preparation. We recommend using a maximum filter size of 100 μm to prevent clogging of fluidics. However, elongated pollen with large size in the longest direction that can pass the filter can still be measured (*c*. 120 μm).
      ▪ Flow speed: The flow speed is set to ‘Low’; otherwise, image quality is not sufficient to derive accurate cell size estimates.
      ▪ Acquired particle number: We aim to collect data of 5000 particles if possible or restrict the time to a maximum of 10 min.
      ▪ Pixel resolution
        ×20 objective at ‘Low’ speed 1 × 1 μm.
        ×40 objective at ‘Low’ speed 0.5 × 0.5 μm.
**# RECOMMENDATION** – A minimum of 50 μl sample volume should be prepared in the autosampler mode, since lower volumes might impede the fluidics system. In the manual mode, the minimal sample volume can be 30 μl. If the manual mode is used, it is recommended to sufficiently vortex the sample before being measured but avoid air bubbles in the sample, which can affect stable laminar flow.
**# RECOMMENDATION** – A threshold on size should be used during image collection in order to reduce data storage of nonpollen images (i.e. small debris particles).
**Δ CRITICAL** – If size threshold is set too high, a systematic exclusion of potentially relevant pollen might happen. For single species samples, the threshold can be more restricted in comparison with mixed species samples or environmental samples with an unknown pollen composition.
    ○ Further information on software, settings and performance of measurements, etc., is available in the manufacturer's instructions.
    ○ Expected data storage space
      ▪ Measurements with images of 5000 acquired particles usually result in a storage space of *c*. 100–500 Mb (rif‐file). Conversion of files into cif‐ and daf‐files during data analysis will increase disk space by a factor of *c*. 2.
    ○ Metadata storage
      ▪ Metadata documentation is very important for a FAIR data management practice and a sustainable data life cycle.
**# RECOMMENDATION** – It is recommended to store all relevant information on sample collection and processing, instrument details, measurement and data analysis templates, purpose of the experiment, according to the MIFlowCyt standard (Lee *et al*., 2008; Spidlen *et al*., 2012).
  - **Data export**
    ○ Export as fcs‐file or txt‐file via the Ideas software
    ○ Export image features via R environment (Demont *et al*., 2024) (see supplementary example script)

## Supporting information


**Fig. S1** Size comparison of commercially ordered and field collected pollen.
**Fig. S2** Size (‘Length’‐feature) of L20 latex beads estimated by imaging flow cytometry using different mask settings.

**Table S1** Measured pollen size using imaging flow cytometry and metadata, including taxonomic information, number of collected years and orientation of pollen per species.
**Table S2** Pearson's *r* correlation coefficient between imaging flow cytometry size features and literature values.
Please note: Wiley is not responsible for the content or functionality of any Supporting Information supplied by the authors. Any queries (other than missing material) should be directed to the *New Phytologist* Central Office.

## Data Availability

The data and code supporting the results of this study are available at doi: 10.5281/zenodo.14866145

## References

[nph70070-bib-0001] Ackerman JD . 2000. Abiotic pollen and pollination: ecological, functional, and evolutionary perspectives. In: Dafni A , Hesse M , Pacini E , eds. Pollen and pollination. Vienna, Austria: Springer Vienna, 167–185.

[nph70070-bib-0002] Allen GP , Hodgson RM , Marsland SR , Flenley JR . 2008. Machine vision for automated optical recognition and classification of pollen grains or other singulated microscopic objects. In: 2008 15th International Conference on Mechatronics and Machine Vision in Practice. Auckland, New Zealand: IEEE, 221–226. doi: 10.1109/MMVIP.2008.4749537.

[nph70070-bib-0003] Aloisi I , Cai G , Tumiatti V , Minarini A , del Duca S . 2015. Natural polyamines and synthetic analogs modify the growth and the morphology of *Pyrus communis* pollen tubes affecting ROS levels and causing cell death. Plant Science 239: 92–105. 26398794 10.1016/j.plantsci.2015.07.008

[nph70070-bib-0004] Altman DG , Bland JM . 1983. Measurement in medicine: the analysis of method comparison studies. Journal of the Royal Statistical Society Series D: The Statistician 32: 307.

[nph70070-bib-0005] Barnes CM , Power AL , Barber DG , Tennant RK , Jones RT , Lee GR , Hatton J , Elliott A , Zaragoza‐Castells J , Haley SM *et al*. 2023. Deductive automated pollen classification in environmental samples via exploratory deep learning and imaging flow cytometry. New Phytologist 240: 1305–1326. 37678361 10.1111/nph.19186 PMC10594409

[nph70070-bib-0006] Bell BA , Bishop TH , Fletcher WJ , Ryan P , Ilmen R . 2018. *Cedrus atlantica* pollen morphology and investigation of grain size variability using laser diffraction granulometry. Palynology 42: 339–353.

[nph70070-bib-0007] Beug H‐J . 2015. Leitfaden der Pollenbestimmung für Mitteleuropa und angrenzende Gebiete, 2^nd^ edn. München, Germany: Verlag Dr. Friedrich Pfeil.

[nph70070-bib-0008] Bland JM , Altman DG . 1986. Statistical methods for assessing agreement between two methods of clinical measurement. Lancet 327: 307–310. 2868172

[nph70070-bib-0009] Bolinder K , Niklas KJ , Rydin C . 2015. Aerodynamics and pollen ultrastructure in *Ephedra* . American Journal of Botany 102: 457–470. 25784479 10.3732/ajb.1400517

[nph70070-bib-0010] Borrell JS . 2012. Rapid assessment protocol for pollen settling velocity: implications for habitat fragmentation. Bioscience Horizons 5: hzs002.

[nph70070-bib-0011] Cariñanos P , Marinangeli F . 2021. An updated proposal of the potential allergenicity of 150 ornamental trees and shrubs in Mediterranean cities. Urban Forestry & Urban Greening 63: 127218.

[nph70070-bib-0012] Chaturvedi M , Datta K , Nair PKK . 1998. Pollen morphology of *Oryza* (Poaceae). Grana 37: 79–86.

[nph70070-bib-0013] Cruden RW . 2000. Pollen grains: why so many? Plant Systematics and Evolution 222: 143–165.

[nph70070-bib-0014] Cruden RW , Lyon DL . 1985. Correlations among stigma depth, style length, and pollen grain size: do they reflect function or phylogeny? Botanical Gazette 146: 143–149.

[nph70070-bib-0015] Culley TM , Weller SG , Sakai AK . 2002. The evolution of wind pollination in angiosperms. Trends in Ecology & Evolution 17: 361–369.

[nph70070-bib-0016] Dbouk T , Visez N , Ali S , Shahrour I , Drikakis D . 2022. Risk assessment of pollen allergy in urban environments. Scientific Reports 12: 21076. 36473878 10.1038/s41598-022-24819-w PMC9727162

[nph70070-bib-0017] Demont Y , Stoll G , Kroemer G , Marolleau JP , Garçon L . 2024. ifc: an R package for imaging flow cytometry . INSERM, UPD, CHU Amiens. [WWW document] URL https://github.com/gitdemont/IFC. [accessed 24 August 2025].

[nph70070-bib-0018] Dominical V , Samsel L , McCoy JP . 2017. Masks in imaging flow cytometry. Methods 112: 9–17. 27461256 10.1016/j.ymeth.2016.07.013 PMC5205551

[nph70070-bib-0019] Dunker S . 2020. *Device for automatic species analysis and method for carrying out the same* (US020200278300A1, EP000003692357A1). Deutsches Patent‐ und Markenamt.

[nph70070-bib-0020] Dunker S , Boyd M , Durka W , Erler S , Harpole WS , Henning S , Herzschuh U , Hornick T , Knight T , Lips S *et al*. 2022. The potential of multispectral imaging flow cytometry for environmental monitoring. Cytometry Part A: The Journal of the International Society for Analytical Cytology 101: 782–799. 35670307 10.1002/cyto.a.24658

[nph70070-bib-0021] Dunker S , Motivans E , Rakosy D , Boho D , Mäder P , Hornick T , Knight TM . 2021. Pollen analysis using multispectral imaging flow cytometry and deep learning. New Phytologist 229: 593–606. 32803754 10.1111/nph.16882

[nph70070-bib-0022] Durka W , Michalski SG . 2012. Daphne: a dated phylogeny of a large European flora for phylogenetically informed ecological analyses. Ecology 93: 2297.

[nph70070-bib-0023] Fitter AH , Peat HJ . 1994. The ecological flora database. Journal of Ecology 82: 415–425.

[nph70070-bib-0024] Friedman J , Barrett SCH . 2009. Wind of change: new insights on the ecology and evolution of pollination and mating in wind‐pollinated plants. Annals of Botany 103: 1515–1527. 19218583 10.1093/aob/mcp035 PMC2701749

[nph70070-bib-0025] Gill JS , Beevers DG , Zezulka AV , Davies P . 1985. Relation between initial blood pressure and its fall with treatment. Lancet 325: 567–569. 10.1016/s0140-6736(85)91219-x 2857912

[nph70070-bib-0026] Grega L , Anderson S , Cheetham M , Clemente M , Colletti A , Moy W , Talarico D , Thatcher SL , Osborn JM . 2013. Aerodynamic characteristics of saccate pollen grains. International Journal of Plant Sciences 174: 499–510.

[nph70070-bib-0027] Halbritter H , Ulrich S , Grímsson F , Weber M , Zetter R , Hesse M , Buchner R , Svojtka M , Frosch‐Radivo A . 2018. Illustrated pollen terminology. Cham, Switzerland: Springer.

[nph70070-bib-0028] Hall JA , Walter GH . 2011. Does pollen aerodynamics correlate with pollination vector? Pollen settling velocity as a test for wind versus insect pollination among cycads (Gymnospermae: Cycadaceae: Zamiaceae). Biological Journal of the Linnean Society 104: 75–92.

[nph70070-bib-0029] Hayat MQ , Ashraf M , Khan MA , Yasmin G , Shaheen N , Jabeen S . 2009. Phylogenetic analysis of *Artemisia* L. (Asteraceae) based on micromorphological traits of pollen grains. African Journal of Biotechnology 8: 6561–6568.

[nph70070-bib-0030] Hofmann F , Otto M , Wosniok W . 2014. Maize pollen deposition in relation to distance from the nearest pollen source under common cultivation – results of 10 years of monitoring (2001 to 2010). Environmental Sciences Europe 26: 24.

[nph70070-bib-0031] Hofmann P , Clark A , Hoffmann P , Chatzinotas A , Harpole WS , Dunker S . 2021. Beyond nitrogen: phosphorus – estimating the minimum niche dimensionality for resource competition between phytoplankton. Ecology Letters 24: 761–771. 33590958 10.1111/ele.13695

[nph70070-bib-0032] Holt KA , Bebbington MS . 2014. Separating morphologically similar pollen types using basic shape features from digital images: a preliminary study. Applications in Plant Sciences 2: apps.1400032. doi: 10.3732/apps.1400032. 25202650 PMC4141716

[nph70070-bib-0033] Hornick T , Richter A , Harpole WS , Bastl M , Bohlmann S , Bonn A , Bumberger J , Dietrich P , Gemeinholzer B , Grote R *et al*. 2022. An integrative environmental pollen diversity assessment and its importance for the Sustainable Development Goals. Plants, People, Planet 4: 110–121.

[nph70070-bib-0034] Johansen B , von Bothmer R . 1994. Pollen size in *Hordeum* L.: correlation between size, ploidy level, and breeding system. Sexual Plant Reproduction 7: 259–263.

[nph70070-bib-0035] Joly C , Barillé L , Barreau M , Mancheron A , Visset L . 2007. Grain and annulus diameter as criteria for distinguishing pollen grains of cereals from wild grasses. Review of Palaeobotany and Palynology 146: 221–233.

[nph70070-bib-0036] Kattge J , Bönisch G , Díaz S , Lavorel S , Prentice IC , Leadley P , Tautenhahn S , Werner GDA , Aakala T , Abedi M *et al*. 2020. TRY plant trait database – enhanced coverage and open access. Global Change Biology 26: 119–188. 31891233 10.1111/gcb.14904

[nph70070-bib-0037] Kattge J , Díaz S , Lavorel S , Prentice IC , Leadley P , Bönisch G , Garnier E , Westoby M , Reich PB , Wright IJ *et al*. 2011. TRY – a global database of plant traits. Global Change Biology 17: 2905–2935.

[nph70070-bib-0038] Lau T‐C , Stephenson A . 1994. Effects of soil phosphorus on pollen production, pollen size, pollen phosphorus content, and the ability to sire seeds in *Cucurbita pepo* (Cucurbitaceae). Sexual Plant Reproduction 7: 215–220.

[nph70070-bib-0039] Lee JA , Spidlen J , Boyce K , Cai J , Crosbie N , Dalphin M , Furlong J , Gasparetto M , Goldberg M , Goralczyk EM *et al*. 2008. MIFlowCyt: the minimum information about a flow cytometry experiment. Cytometry Part A: The Journal of the International Society for Analytical Cytology 73: 926–930. 18752282 10.1002/cyto.a.20623 PMC2773297

[nph70070-bib-0040] Leslie AB . 2010. Flotation preferentially selects saccate pollen during conifer pollination. New Phytologist 188: 273–279. 20579290 10.1111/j.1469-8137.2010.03356.x

[nph70070-bib-0041] Lu K‐Q , Xie G , Li M , Li JF , Trivedi A , Ferguson DK , Yao YF , Wang YF . 2018. Dataset of pollen morphological traits of 56 dominant species among desert vegetation in the eastern arid central Asia. Data Brief 18: 1022–1046. 29900271 10.1016/j.dib.2018.03.122 PMC5996618

[nph70070-bib-0042] Lu X , Ye X , Liu J . 2022. Morphological differences between anemophilous and entomophilous pollen. Microscopy Research and Technique 85: 1056–1064. 34726304 10.1002/jemt.23975

[nph70070-bib-0043] Mäkelä EM . 1996. Size distinctions between *Betula* pollen types – a review. Grana 35: 248–256.

[nph70070-bib-0044] Moore J , Kunis S , Grüning B , Blank‐Burian M , Mallm J‐P , Stöter T , Zuschratter W , Figge MT , Kreshuk A , Tischer C *et al*. 2024. NFDI4BIOIMAGE – National research data infrastructure for microscopy and bioimage analysis. *Zenodo*. doi: 10.5281/zenodo.13168693.

[nph70070-bib-0045] Müller F , Ritz CM , Welk E , Wesche K . 2021. Rothmaler – Exkursionsflora von Deutschland. Gefäßpflanzen: Grundband. Berlin, Heidelberg, Germany: Springer Berlin Heidelberg.

[nph70070-bib-0046] Münkemüller T , Lavergne S , Bzeznik B , Dray S , Jombart T , Schiffers K , Thuiller W . 2012. How to measure and test phylogenetic signal. Methods in Ecology and Evolution 3: 743–756.

[nph70070-bib-0047] Rohde E . 1959. Überprüfung und Ausbau der Getreide‐Pollenanalyse . Unveröff, Staatsexamensarbeit Göttingen.

[nph70070-bib-0048] Schwendemann AB , Wang G , Mertz ML , McWilliams RT , Thatcher SL , Osborn JM . 2007. Aerodynamics of saccate pollen and its implications for wind pollination. American Journal of Botany 94: 1371–1381. 21636505 10.3732/ajb.94.8.1371

[nph70070-bib-0049] Senghas K , Seybold S . 2003. Schmeil – Fitschen: Flora von Deutschland und angrenzender Länder – Ein Buch zum Bestimmen der wildwachsenden und häufig kultivierten Gefäßpflanzen, 92, durch. Wiebelsheim, Germany: Quelle & Meyer Verlag.

[nph70070-bib-0050] Sork VL , Davis FW , Smouse PE , Apsit VJ , Dyer RJ , Fernandez‐M JF , Kuhn B . 2002. Pollen movement in declining populations of California Valley oak, *Quercus lobata*: where have all the fathers gone? Molecular Ecology 11: 1657–1668. 12207717 10.1046/j.1365-294x.2002.01574.x

[nph70070-bib-0051] Sótonyi P , Szabó Z , Nyéki J , Benedek P , Soltész M . 2000. Pollen morphology of fruit species. International Journal of Horticultural Science 6: 49–57.

[nph70070-bib-0052] Spidlen J , Breuer K , Brinkman R . 2012. Preparing a minimum information about a flow cytometry experiment (MIFlowCyt) compliant manuscript using the International Society for Advancement of Cytometry (ISAC) FCS file repository (FlowRepository.org). Current Protocols in Cytometry 61: 1–26. 10.1002/0471142956.cy1018s61 22752950

[nph70070-bib-0053] Stebler T . 2022. Pollenatlas, Pollen‐Wiki . [WWW document] URL https://pollen.tstebler.ch/MediaWiki/index.php?title=Pollenatlas [accessed 24 August 2022].

[nph70070-bib-0054] Theuerkauf M , Couwenberg J . 2022. Pollen productivity estimates strongly depend on assumed pollen dispersal II: extending the ERV model. The Holocene 32: 1233–1250.

[nph70070-bib-0055] Theuerkauf M , Nehring E , Gillert A , Bodien PM , Hein M , Urban B . 2024. First automatic size measurements for the separation of dwarf birch and tree birch pollen in MIS 6 to MIS 1 records from Northern Germany. Ecology and Evolution 14: 1–17. 10.1002/ece3.11510 PMC11176728 38882530

[nph70070-bib-0056] Walther F , Hofmann M , Rakosy D , Plos C , Deilmann TJ , Lenk A , Römermann C , Harpole WS , Hornick T , Dunker S Multispectral imaging flow cytometry for spatio‐temporal pollen trait variation measurements of insect‐pollinated plants. Accepted for publication in *Cytometry Part A*. 10.1002/cyto.a.24932 40200568

[nph70070-bib-0057] Wei C , Jardine PE , Gosling WD , Hoorn C . 2023. Is Poaceae pollen size a useful proxy in palaeoecological studies? New insights from a Poaceae pollen morphological study in the Amazon. Review of Palaeobotany and Palynology 308: 104790.

[nph70070-bib-0058] Wei C , Jardine PE , Mao L , Mander L , Li M , Gosling WD , Hoorn C . 2024. Grass pollen surface ornamentation is diverse across the phylogeny: evidence from northern South America and the global literature. Journal of Systematics and Evolution 62: 687–701.

[nph70070-bib-0059] Wickham H . 2016. ggplot2: elegant graphics for data analysis. New York, NY, USA: Springer‐Verlag.

[nph70070-bib-0060] Wickham H , François R , Henry L , Müller K . 2022. dplyr: a grammar of data manipulation . R package v.1.0.10. [WWW document] URL https://CRAN.R‐project.org/package=dplyr

[nph70070-bib-0061] Williams CG . 2010. Long‐distance pine pollen still germinates after meso‐scale dispersal. American Journal of Botany 97: 846–855. 21622450 10.3732/ajb.0900255

[nph70070-bib-0062] Yang S , Zheng Z , Huang K , Zong Y , Wang J , Xu Q , Rolett BV , Li J . 2012. Modern pollen assemblages from cultivated rice fields and rice pollen morphology: application to a study of ancient land use and agriculture in the Pearl River Delta, China. The Holocene 22: 1393–1404.

