# Supplementary material for: High‐throughput assessment of anemophilous pollen size and variability using imaging cytometry
Source: New Phytol. 2025 Mar 28;246(4):1875–88. doi: 10.1111/nph.70070 (PMC12018789; doi:10.1111/nph.70070)
Supplement: Supplementary file 1 — Fig. S1 Size comparison of commercially ordered and field collected pollen. Fig. S2 Size (‘Length’‐feature) of L20 latex beads estimated by imaging flow cytometry using different mask settings. [file NPH-246-1875-s002.pdf]

New Phytologist

***New Phytologist* Supporting Information**

Article title: **High-throughput assessment of anemophilous pollen size and variability using imaging cytometry**

Authors: Thomas Hornick [1, 2], W Stanley Harpole [1, 2, 3], Susanne Dunker[1, 2]

[1]Helmholtz-Centre for Environmental Research (UFZ) Department of Physiological Diversity Permoserstraße 15, 04318 Leipzig DE; [2]German Centre for Integrative Biodiversity Research (iDiv) Halle-Jena-Leipzig Puschstraße 4, 04103 Leipzig DE; [3]Martin-Luther-Universität Halle-Wittenberg, Naturwissenschaftliche Fakultät I – Biowissenschaften, 06099 Halle (Saale) DE

Author(s) for correspondence:

Thomas Hornick, Email: thomas.hornick@ufz.de;

Susanne Dunker, Email: susanne.dunker@ufz

ORCID

Thomas Hornick 0000-0003-0280-9260

W Stanley Harpole 0000-0002-3404-9174

Susanne Dunker 0000-0001-7276-776X

Article acceptance date: 24 February 2025

New Phytologist

The following Supporting Information is available for this article:

Fig. S1 A) Size of a set of 16 commercially ordered (CP) and field collected pollen (FP) that was measured using IFC. Size of CP and FP is comparable by visual inspection ($y = 0.98x + 0.01$; $df = 14$; $R^2_{adj} = 0.95$; $p < 0.001$). Black line indicates line of equality. **B)** Bland-Altman-Plot representing the deviation between CP and FP (µm) across the pollen size range at species level.

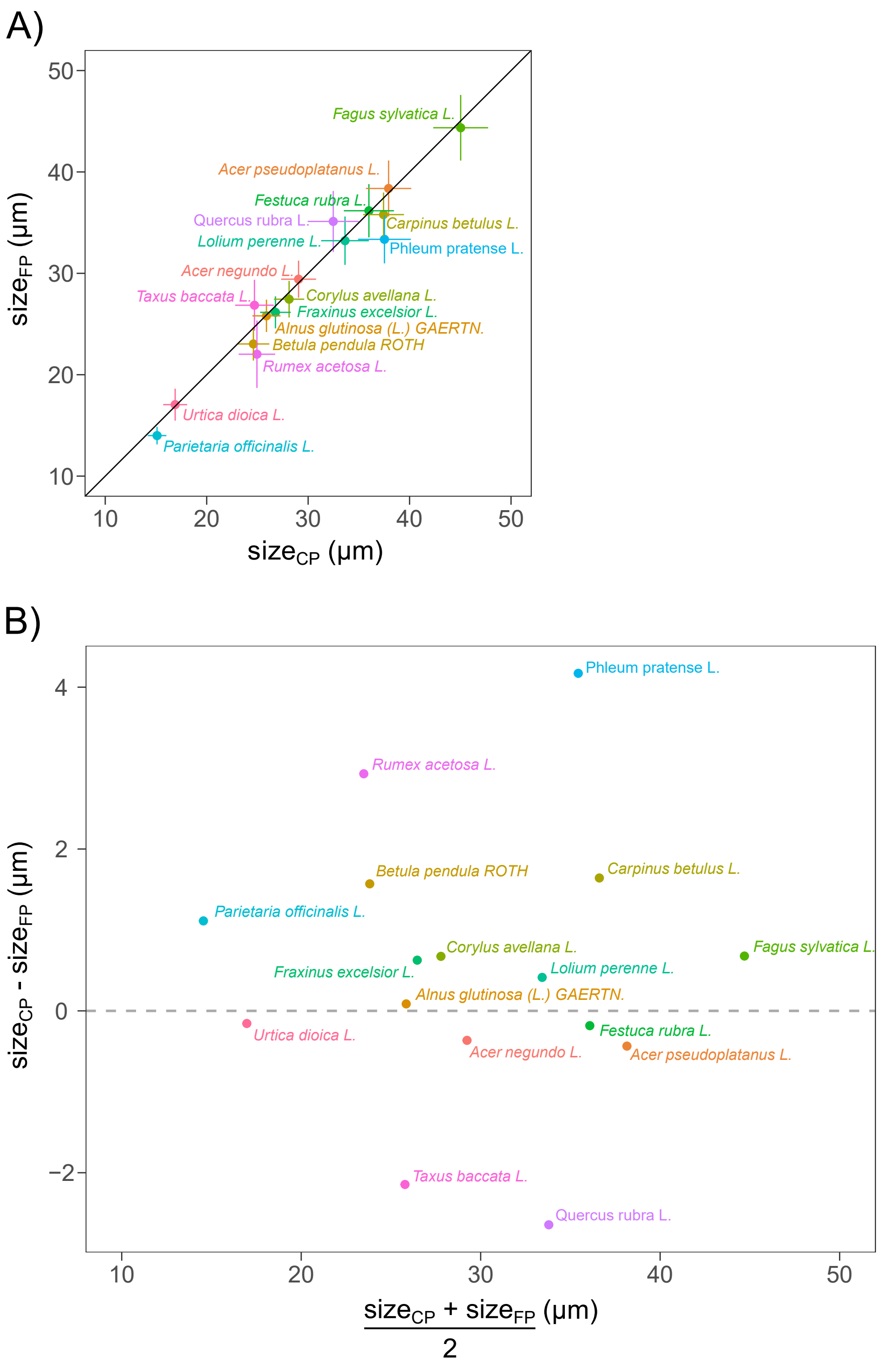

Fig. S2 Size ('Length'-feature) of L20 latex beads (Coulter Corporation, Miami, FL, USA) estimated by IFC using different mask settings. L20 latex beads have a certified modal size of 19.98 µm (red line) traceable to NIST standard. The default masks of the brightfield channels on camera one (Ch01) and two (Ch09) largely overestimate particle size. The optimized Adaptive Erode (AE) mask with a cutoff of 95 on an Object mask of Ch09, which was used for estimation of pollen size in this study, recovered the modal size of the L20 latex beads. Bin width represent the lowest resolution of the 'Length' size-feature based on pixel size of the used **A)** 20x objective (1 µm) and **B)** 40x objective (0.5 µm).

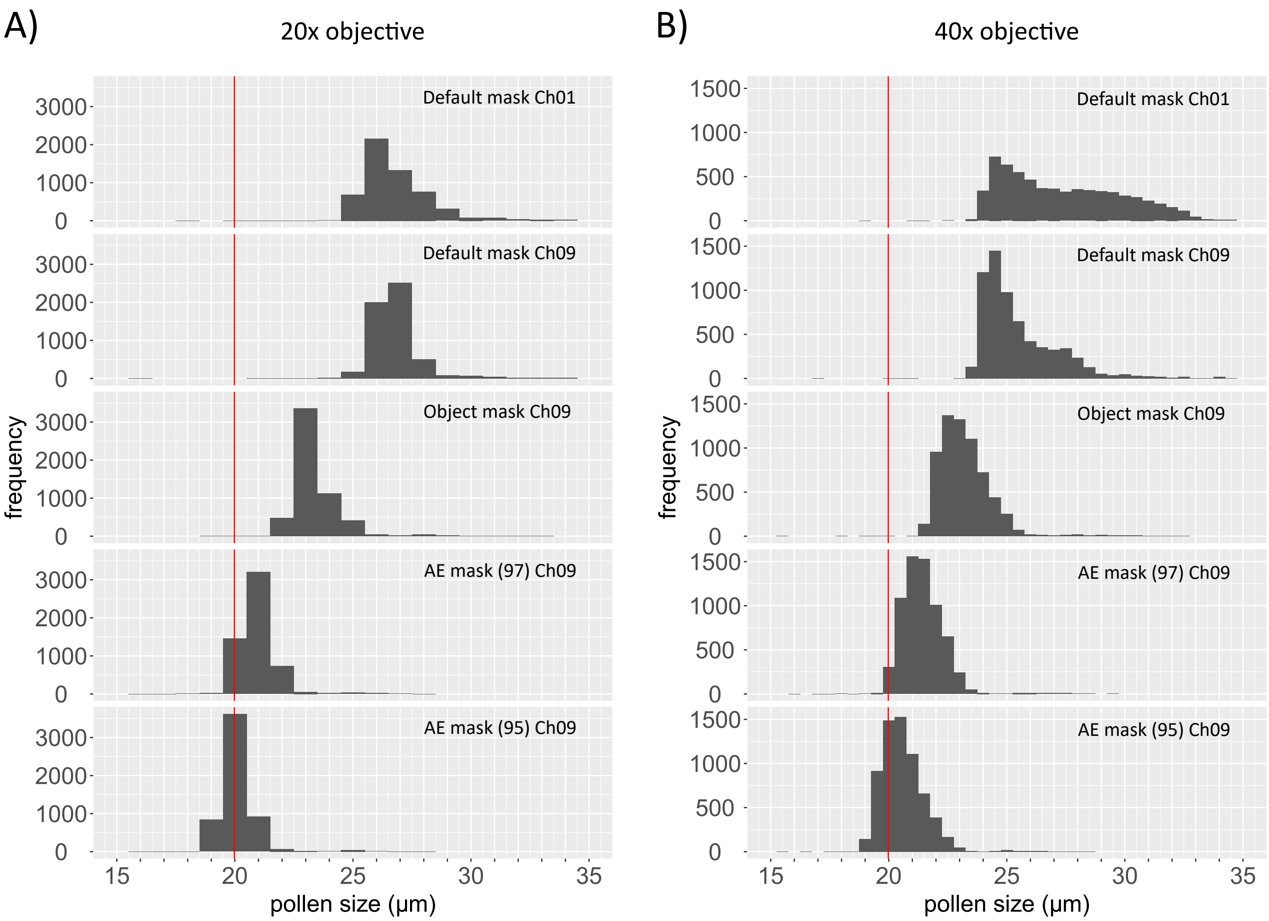

Table S1 A) Plant species, taxonomic order and family level and the orientation of pollen for which the pollen size was estimated (HQ – "spherical" pollen/no specific orientation considered; HQEUQ – equatorial view (oblate and/or "elongated" pollen). For 16 species pollen was ordered from commercially suppliers (ALL - Allergon AB (Thermo Fisher Scientific, Ängelholm, Sweden), BON - Bonapol, a.s., České Budějovice, Czechia). For each species the number of pollen is listed that was measured from field collected samples as well as reference material. For field collected pollen the number of individual plants and years in which the pollen was collected is listed. **B)** Mean and standard deviation (SD) of true biological replicates (mean pollen size of individual plants). Mean pollen size, standard deviation, standard error, confidence intervals (90%, 75%), quantiles (Q1, Q3) and interquantile ranges (IQR) per species calculated for **C)** all measured pollen including ordered reference material and **D)** all field collected pollen. **E)** Summary of literature values from Beug (2015) including data from Rohde (1959), the Pollen-Wiki (http://pollen.tstebler.ch) as well as data from the Ecological Flora Database (Fitter and Peat 1994) as contributed to the TRY-database (Kattge et al. 2020) that were used in this study.

The Table S1 is available as separate Excel-file. It is also part of the data, code and tables available at the Zenodo repository (doi is added in the "Data Availability Statement").

Table S2 Pearson´s r correlation coefficient between IFC size features and literature values from Beug (2015) including data from Rohde (1959) (d*f* = 66, p < .001), data from the Pollen-Wiki (http://pollen.tstebler.ch) (d*f* = 36, p < .001) as well as data from the Ecological Flora Database (Fitter and Peat 1994) as contributed to the TRY-database (Kattge et al. 2020) (df = 36, p < .001). The correlations between MIFC data and data from literature compare as good as data between literature sources (Beug ~ Pollen-Wiki: d*f* = 30, p < .001; Beug ~ TRY: d*f* = 34, p < .001; Pollen-Wiki ~ TRY: d*f* = 19, p < .001). The feature estimates are based on an Adaptive Erode (AE) mask with a cutoff of 95 on an Object mask of Channel 09.

**Feature/literature source**	**Beug (2015)**	**Pollen-Wiki**	**TRY**
‘Diameter’	0.95	0.97	0.91
‘Height’	0.96	0.97	0.91
‘Length’	0.96	0.97	0.91
‘Major Axis’	0.96	0.97	0.91
‘Thickness Max’	0.88	0.93	0.82
‘Width’	0.92	0.95	0.85
**Beug (2015)**		0.96	0.87
**Pollen-Wiki**			0.97
